# Modern Fire-Resistant Fabrics—Requirements for Durability of Materials After Washing After a Fire

**DOI:** 10.3390/ma19010044

**Published:** 2025-12-22

**Authors:** Anna Rabajczyk, Maria Zielecka, Michał Chmiel

**Affiliations:** Scientific and Research Centre for Fire Protection-National Research Institute, Nadwiślańska 213, 05-420 Józefów, Poland; mzielecka@cnbop.pl (M.Z.); mchmiel@cnbop.pl (M.C.)

**Keywords:** fire-resistant fabric, protective clothing, cleaning, durability

## Abstract

Developments in the textile industry occur both as a consequence of increased awareness among users and various requirements in terms of human and environmental safety. Modifications are aimed at improving performance parameters, using natural substances, moving away from synthetic materials, and improving ergonomics. In order to achieve this, various fibre-production techniques are used, as is the addition of substances, including nanosubstances, into the structure or onto the surface of a given material. In the case of fire-resistant fabrics, which primarily must meet thermal protection requirements, efforts are also being made to reduce weight and eliminate harmful chemicals (e.g., polycyclic aromatic hydrocarbons PAHs), and to create smart materials with sensors. However, it is necessary to further develop not only the materials themselves but also cleaning and decontamination techniques that will allow the fire resistance parameters that have been developed to be maintained.

## 1. Introduction

Recent decades have seen intensive developments in textile technology aimed at improving the safety of users of protective clothing when exposed to high temperatures and fire. Increased awareness of fire hazards and stricter safety standards in many industrial sectors, emergency services, and the military have contributed to increased demand for modern materials with fire-resistant properties. In addition to the requirements for effective thermal protection, ergonomic aspects such as comfort of use, breathability, and resistance to repeated washing and dry cleaning have also become crucial.

An example of a solution worth considering is the development of so-called ‘smart materials’, which incorporate sensors, such as a temperature nanosensor, into the structure of a fabric. These materials allow for the monitoring of both the environmental quality at the fire scene and the comfort of the wearer [1].

Requirements for fire-resistant fabrics and clothing designed for firefighting services must therefore be based on the universally recognized human–technology–environment (H-T-E) system, which considers three aspects: the person, the rescue equipment, and the location of operation. The fire environment, as a workplace for firefighters, for example, is characterized by the impacts of external factors resulting from the specific nature of the incident and the firefighting actions undertaken; the occurrence of stress, generating psychophysical exhaustion; and destructive impacts relating to physicochemical environmental parameters, posing a threat to health and life. A wide range of hazard factors includes the impacts of, among others, a hot microclimate; an environment with increased chemical activity; and an environment with a complex, active interplay of hazard factors, which can even result in loss of life. The increased awareness of these dangers and the emergence of requirements in this area form the basis for the selection of materials and the implementation of changes and modifications [1].

The fire resistance of fabrics can be achieved in two primary ways: by using fibres with inherent flame-retardant properties or by applying appropriate impregnation or chemical treatment to give the fibres non-flammable properties or at least reduce their flammability. Inherently flame-retardant fibres include, among others, aromatic polyamide fibres, known as aramids (e.g., Nomex^®^, Kevlar^®^); polybenzimidazole fibres; modified acrylic fibres made of acrylonitrile, known as modacrylics; polysulfide phenylene fibres, known as PPS; and polymetaphthalimide fibres, known as meta-aramids. All of the abovementioned fibres, due to their chemical structure, are characterised by high thermal resistance and limited melting when in contact with flames [2,3]. A second group consists of fibres that have undergone finishing treatment with flame retardants, such as phosphorus, nitrogen, or boron compounds, which form protective layers on the surface of the fibres or affect the pyrolysis process when exposed to high temperatures [4].

Despite significant progress in the area of chemical flame-retardant methods for fabrics, the durability of the impregnation remains as one of the main problems. Flame retardants tend to gradually degrade or wash out during washing cycles, and with exposure to UV radiation, dry cleaning, or contact with sweat and moisture [5]. As a result, the protective effect of the material may be significantly reduced after just a handful of cycles of use. For this reason, assessing the durability of impregnation after washing and after actual exposure to fire is a key element in research regarding materials intended for protective clothing.

From a normative perspective, the requirements for the flame resistance and thermal resistance of protective fabrics are specified by numerous international standards, including ISO 15025 [6], ISO 11612 [7], EN 469 [8], EN 531 [9], and NFPA 2112 [10]. These standards define both the testing methods and the criteria for classifying fabrics in terms of their behaviour in contact with fire, degree of charring, and burning rate, and in cases of dripping liquids. Increasingly, the durability of protective parameters after washing is also taken into account, reflecting the real-life conditions of use of protective clothing in the work environment.

In recent years, intensive research has been conducted regarding the development of more chemically and physically stable flame-retardant systems. Modern solutions include nanocomposites, coatings using metal nanoparticles and metal oxides, systems based on phosphorus–nitrogen compounds, and sol–gel technologies that enable the permanent anchoring of protective layers on the surface of fibres [11]. At the same time, methods for physicochemical modification of fibre surfaces are being developed to improve the adhesion of impregnants and increase the fabric’s resistance to leaching [12,13].

The aim of this publication is to review and present modern fire-resistant fabrics, with particular emphasis on the durability of their properties after washing and exposure to fire. The paper discusses the current state of knowledge in the area of flame-retardant methods, the characteristics of chemical agents, factors affecting the stability of fibres and protective coatings, and methods for assessing the effectiveness and durability of protective fabrics in laboratory and real-life conditions. This analysis aims to indicate the directions for further research and possibilities for improving the durability of the fire-resistant properties of clothing.

## 2. Method of Conducting the Literature Review

A literature review surveying the last 9 years was performed, considering the following aspects: durability of fire-resistant fabrics and the resistance of fire-retardant fabrics used by firefighters.

Trends in the numbers of publications regarding fire-resistant fabrics for firefighters from 2016 to 2025, based on the data from the Web of Science (Table 1), show an increasing interest in this topic, especially in the area of fire-retardant fabrics used by firefighters.

The abovementioned rising interest in the topic is only further confirmed by the number of citations presented in the table, a factor which has been significantly growing since 2019.

**Figure 1:**
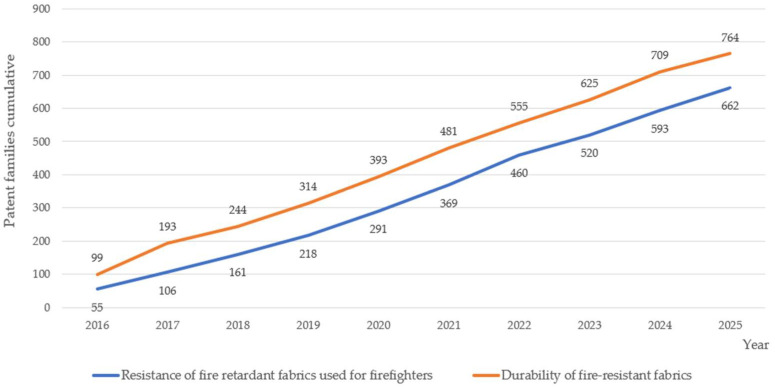
Number of patent families relating to the durability of fire-resistant fabrics and resistance of fire-resistant fabrics used by firefighters (based on the Espacenet search).

The research also included Espacenet, Patentscope, and Google Patents, and searched the years 2016–2025 (until 31 October 2025), which resulted in the identification of selected patents relevant to the subject. For example, in the Espacenet search, there were 764 results found for the keywords ‘durability of fire-resistant fabrics’ and 662 results for the keywords ‘resistance of fire-resistant fabrics used for firefighters’; see Figure 1.

In the Espacenet search engine, a patent family is defined as ‘including all documents with exactly the same priority or combination of priorities’.

Certain patents, selected based on the most promising applications, will be discussed below. This review does not, however, cover the use of flame retardants, especially the halogenated ones. The use of such additives is limited in the countries of the European Union and the USA due to certain legal provisions [e.g., Registration, Evaluation, Authorisation and Restriction of Chemicals (REACH regulation), The Restriction of Hazardous Substances Directive (RoHS Directive)].

## 3. Characteristics of Fire-Resistant Fabrics

Fire-resistant fabrics are widely used in the production of protective clothing for firefighters, petrochemical industry workers, steelworkers, energy sector employees, and the military. They are also used to manufacture everyday products such as upholstery, curtains, furniture, and tents. Intelligent integrations using coatings and membranes, laminating panels, and other selected outer and inner components of clothing create enormous potential in the development of fabrics [14]. Multi-layer laminates are also used in certain high-risk applications, such as firefighting clothing and aircraft interiors. These solutions enable increased fabric protection and better thermal efficiency while ensuring the comfort of use. Two- or three-layer systems allow for the combinations of materials with different properties, such as a system with a flame-retardant outer layer with a moisture-wicking inner layer [15,16]. The protective properties of such materials stem from their ability to delay ignition, limit flame spread, create a charred layer, and minimise heat transfer to the inside of the garment [17,18].

It should be emphasised that the terms ‘fire-retardant’ and ‘flame-resistant’, when used in the context of fabrics, are not interchangeable, as there are key differences between them. Flame-retardant fabrics use flame retardants that, when applied to the fabric, make it resistant to flames. They contain a special finish using such chemical fibres. This coating helps prevent the spread of flames. In addition, they burn much more slowly than their standard, untreated counterparts. Over time, however, the chemicals are destroyed during washing, rendering them ineffective unless the appropriate substances are reapplied [19]. Flame-resistant materials, on the other hand, contain synthetic fibres that are resistant to ignition and subsequently self-extinguish after being exposed to prolonged heat or flame. Because these fabrics are woven from inherently flame-resistant fibres, they are often referred to as IFR (inherently fire-resistant) fabrics. Therefore, from a technical perspective, these fabrics are divided into two primary groups [4,20]:

- Fabrics made from inherently flame-retardant fibres;
- Fabrics impregnated with flame retardants.

The first group includes materials the fire resistance of which is a property resulting from the chemical structure of the fibres, e.g., aramid fibres (Nomex^®^, Kevlar^®^), PBI, modacrylic, polybenzimidazole, or poly(phenylene sulphide). They are characterised by a high softening temperature, low thermal conductivity, and thermal stability, even above 400 °C [21]. The second group is based on standard fibres (e.g., cotton, viscose, polyester, and polyamide) that undergo chemical treatment, providing them with flame-retardant properties [22]. However, the degree of fire resistance of a given fabric varies depending on the fibres used during the fabric production process [23,24].

Another classification takes into consideration the material’s resistance to washing; according to this measure, there are four types: disposable flame-retardant fabrics, semi-washable flame-retardant fabrics, washable flame-retardant fabrics, and permanently flame-retardant fabrics [25]. They all possess different characteristics and have different applications. For example, disposable flame-retardant fabrics can provide temporary fire protection in the event of a fire, but their fire resistance is significantly reduced after washing in water. They can therefore be used to manufacture, for example, evacuation suits and covers for fire-fighting equipment. Semi-washable flame-retardant fabrics can be used, in turn, in the production of workwear used for a specific period of time, or temporary covers for firefighting equipment. Their effectiveness depends on the number of washes—the more often the fabric is cleaned, the weaker its fire-resistant properties become [25].

Repeated washing, usually more than 50 times, without affecting the fire-resistant properties, is possible in the case of flame-retardant fabrics suitable for washing. They are characterised by good wash resistance, which makes them suitable for applications requiring long-term fire protection and frequent cleaning, such as fire-resistant workwear, fire-resistant bedding, etc. Permanently flame-retardant fabrics possess the highest resistance, with their fire-resistant properties remaining stable throughout their entire service life and withstanding even frequent washing. Flame retardants are usually added to them during the fibre-production process in order to integrate the retardants into the fibre itself [25].

## 4. Flame Retardant Fabrics

Fabric modifications are carried out using various methods, with increasing emphasis being placed on the development of solutions and technologies that also meet environmental protection requirements, such as the layer-by-layer self-assembly method, the sol–1gel method, the microencapsulation method, and the plasma method [26,27,28] (Table 2).

One of the methods most commonly used to achieve flame retardancy in fabrics is impregnation with flame retardants. This involves the application of substances which, when in contact with fire, limit the combustion process through one or more mechanisms [36,37,38]:

- Formation of a charred layer (condensation effect);
- Release of non-combustible gases (gas phase effect);
- Thermal decomposition with the release of products that inhibit the chain reaction of combustion;
- Restriction of oxygen access to the fibre surface.

The most commonly used flame retardants include the following:

− Phosphorus compounds—Phosphates, phosphonates, phosphorines, and their derivatives (e.g., THPC, TEPAP), which promote the formation of a charred layer and are commonly used for impregnating cellulose fabrics.
− Nitrogen compounds—Often used in combination with phosphorus, increase thermal stability and synergistically support the carbonisation process.
− Boron and silicon compounds—Act by forming a glassy protective layer that limits oxygen access.
− Metal oxide nanoparticles (TiO_2_, SiO_2_, Al_2_O_3_, ZnO)—Used in modern multi-layer coatings, increase thermal resistance and improve the adhesion of the impregnating agent to the fibres [39]. Due to strong chemical bonds with the fibre surface, they exhibit increased resistance to leaching compared to traditional flame retardants. Promising results have also been obtained for aerogel fibres based on ultra-light aramid nanofibres.

Sol–gel systems and hybrid coatings, in which organic and inorganic components form a durable protective network on the fabric surface, are becoming increasingly popular [40]. Due to their strong chemical bonds with the fibre surface, they exhibit increased resistance to leaching, compared to traditional flame retardants. Promising results have also been obtained for aerogel fibres based on ultra-light aramid nanofibers [41,42].

## 5. Key Factors Affecting Fibre Durability After Fire and Washing

**Figure 2:**
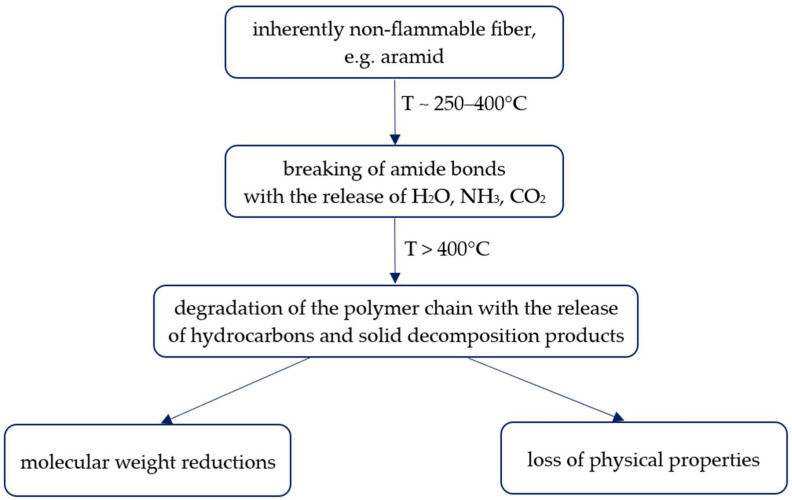
Thermal degradation of aramid fibre.

Thermal degradation occurs in both inherently flame-retardant fibres and fibres containing flame retardants. Thermal degradation of aramid fibres occurs through two main mechanisms: chain depolymerisation and amide bond cleavage reaction [43]. As the temperature rises, the amide bonds break down, leading to the release of gaseous decomposition products such as water, ammonia, and carbon dioxide. At higher temperatures, the chain breaks down into smaller fragments; see Figure 2.

It should be emphasised that higher temperatures result in faster degradation. In addition, prolonged exposure to high temperatures accelerates the degradation process, similarly to the presence of oxygen, which significantly intensifies the oxidation process. Research into the influence of macrostructure and stability on the flammability of nonwovens conducted by Kerekes et al. [44] has shown that the flammability of a fabric is determined both by the properties of the fibre (in this case, oxidised fibre—microstructure) and by various weave structures of the fabric (macrostructure).

Exposure to high temperatures or direct contact with flames also causes thermal decomposition of flame retardants in flame-retardant fibres, leading to their irreversible loss. Furthermore, the durability of flame retardants depends on the type of fibre, the application method used, and the conditions of use and cleaning, as well as the properties of the impregnating agent itself. During washing, hydrolysis, leaching, and chemical deactivation processes occur, leading to a reduction in the flame-retardant content in the fabric structure. In the case of cellulose materials (e.g., cotton), water and detergents cause phosphates and ammonium compounds to gradually leach. In addition, factors such as the pH of the washing solution, temperature, water hardness, and the presence of softeners can accelerate the degradation of the impregnants [45,46].

Therefore, the durability assessment should include both post-washing tests and post-simulated fire or heat exposure tests [47].

## 6. Methods for Assessing the Durability of Fabrics

The durability and effectiveness of inherently flame-retardant fibres is assessed by determining whether the fibres have undergone mechanical degradation or contamination, e.g., with flammable dirt or unrinsed detergent. Fire-resistant impregnated fabrics are, in turn, tested to confirm that the impregnants have not been washed out. The assessment of fabric non-flammability is carried out in two stages: the preparatory stage, involving an ageing simulation (conditioning), and the verification stage, consisting of fire tests after the ageing, including flame spread, heat transfer, and thermal testing. Both stages are carried out using standardised research methods. The most commonly used methods are the following:

- AATCC 61 [48] or ISO 6330 [49] Test—Simulation of washing processes to determine the durability of impregnation;
- ISO 15797 [50]—Simulation of industrial washing processes to test resistance to aggressive chemicals and high temperatures;
- ISO 15025 [6], ASTM D6413 [51] Test—Testing the behaviour of fabric in contact with flame (vertical flame test);
- ISO 11612 [7] Test—Assessment of material resistance to convective heat and thermal radiation;
- Thermogravimetric Analysis (TGA)—Determining the thermal stability of a material;
- FTIR and SEM-EDS—Analytical techniques for assessing the presence of the impregnating agent and its changes after washing;
- ISO 9151 [52]/ ISO 6942 [53]—A test method for comparing the heat transfer through materials or assemblies of materials;
- ISO 13506 [54]—Testing of entire suits using an instrumental manikin to predict burn injuries.

Mechanical verification to determine the durability of a certain fire-retardant fabric that has undergone prior conditioning appears to be equally important. In this regard, the testing methods specified in the following standards are used:

- ISO 13937-2 [55]—Tear strength test;
- ISO 13934-1 [56]—Tensile strength test.

Additionally, confirmation of dimensional stability was achieved by assessing whether the fabric had shrunk by more than 3%.

The results of these tests allow researchers to determine the decrease in protective effectiveness after a certain number of washing cycles, as well as analyse changes in the chemical structure and morphology of the fibres.

Verification of a material’s quality is particularly important in the case of special clothing such as firefighter uniforms. Materials used for firefighters’ clothing undergo numerous laboratory tests to ensure that they meet protection requirements, both when new and after exposure to factors associated with use (mechanical wear, washing, high temperatures, UV radiation, contamination, etc.). One example is the mechanical resistance of fabrics and seams in special clothing, which is critical because firefighters expose their clothing to friction, tears from sharp edges, stress during movement, etc. This parameter is verified from various angles, including abrasion resistance and tensile strength (Table 3).

It should be emphasised that mechanical durability not only concerns the initial values of the material but also focuses on maintaining these parameters over time. Thus, standards often require testing both before and after ageing/processing. For example, EN 469 [8] requires strength, tear, and other tests after exposure to heat or washing.

Another important element subject to verification is comfort of use. This parameter is as important for safety as thermal durability and resistance to chemical agents. Overheated or overburdened firefighters are more prone to accidents. The thermal and ergonomic comfort of clothing may also change over time as a result of wear and tear. Modern fire-resistant materials must not only be fire-resistant but also possess other characteristics important for their area of application.

## 7. The Impacts of Conventional and Advanced Cleaning Techniques on the Durability of Fire-Resistant Fabrics

Fire-resistant fabrics are a key element of protective equipment for workers exposed to high temperatures, flames, and thermal factors. Their functionality stems both from the properties of the fibres (e.g., aramid, modacrylic) and from the flame-retardant finishes used in chemically modified materials (e.g., Proban^®^ cotton). Effective maintenance of protective properties requires proper care, including the use of appropriate washing methods, dry cleaning, and regeneration technologies.

One of the main factors contributing to fabric degradation is the cleaning process. Depending on the type of fibre, the type of flame-retardant finish, and the parameters of the process, cleaning can lead to weakening of the fabric structure, leaching of flame retardants, or deterioration of mechanical and thermal properties of the fabric. Therefore, it is crucial to understand the impacts of both the conventional and the advanced cleaning methods on the durability of FR fabrics in order to enable their proper use and extend their service life.

Water washing remains the most commonly used method for cleaning fire-resistant fabrics. It is relatively inexpensive and widely available but carries the risk of degrading the protective properties of the materials. The key parameters affecting fabric durability are washing temperature, detergent type, bath pH value, and number of cycles.

Tests on Proban^®^ fabric indicate that although its fire-resistant properties are permanent, the physical and chemical parameters of the material, such as tensile strength and surface appearance, gradually deteriorate as a result of repeated washing cycles [80]. After 10 wash cycles under various conditions (baths with different pH and chemical composition), parameters such as the limiting oxygen index (LOI) remained at their initial levels, which indicates a relatively good thermal resistance. However, it has also been shown that a strongly alkaline environment (pH > 10) accelerates the leaching of flame-retardant phosphorus compounds. During standard washing, cotton fibres have also been observed to weaken, a phenomenon which shortens the life of the garment. This process is particularly noticeable when aggressive bleaching detergents are used. Research indicates that the use of mild detergents with a pH between 6.5 and 8.0 preserves the high flame retardancy of Proban^®^ fabrics and cotton fabrics with phosphorus coatings.

As a result, frequent washing with water can reduce the actual service life of protective clothing by as much as 30–40% [81].

An important part of the maintenance of firefighter clothing is the effective removal of polycyclic aromatic hydrocarbons (PAHs), which are produced during rescue operations [82]. These contaminants can settle on protective layers and then migrate into the fabric structure, affecting not only the user’s health but also the properties of the material. Research has shown that washing at 60 °C using multiple rinsing cycles significantly reduces the concentration of PAH on the surface of flame-resistant fabrics as well as in their inner layers [83]. However, intensive washing at higher temperatures may lead to the degradation of moisture and water vapor-resistant membranes, which can reduce the wearing comfort and the mechanical resistance of the clothing. A gentler alternative to aqueous washing is dry cleaning using organic solvents. Solvents effectively remove fatty soiling while minimising fibre swelling and the risk of losing flame retardants [84].

For aramid fabrics, such as Nomex^®^ and Kevlar^®^, manufacturers recommend dry cleaning as one of the safest maintenance methods, provided that bleaches and oxidising agents are avoided, as these can weaken the polymer bonds of the fibres [85].

Methods such as ozone washing—which acts as a disinfectant, freshening agent, and detergent booster while simultaneously enabling washing at lower temperatures—low-water technology, or cleaning in baths with strictly controlled pH allow for reductions in the degradation of flame-resistant fabrics. Due to lower water consumption and the action of low-alkaline detergents, fibres are less susceptible to chemical degradation [86].

Conventional wet cleaning, although widespread, carries several limitations:

− It may cause the leaching of flame retardants from chemically modified fabrics;
− It leads to fibre micro-cracking and loss of structural integrity in cotton fabrics;
− The use of chlorine bleaches can significantly damage aramid fibres, lowering their strength.

In recent years, advanced coatings and surface modifications have been developed to enhance the resistance of flame-resistant fabrics to washing. One example of this is the application of the layer-by-layer method [87], in which fabrics are coated with thin layers of phosphorus compounds and silicates. These coatings exhibit high thermal stability and resistance to repeated detergent washing.

In parallel, research is being conducted on nanoparticulate flame-retardant systems, which, due to strong anchoring within the fibre structure, exhibit high resistance to leaching [88]. A comparative summary of methods for cleaning flame-resistant fabrics is presented in Table 4 below.

The application of advanced cleaning methods, particularly chemical processes and fabric surface reinforcement techniques, can significantly reduce the rate of fibre degradation and the loss of protective properties.

## 8. Directions of Development and Research Perspectives

Contemporary research mainly focuses on nanotechnology and the search for environmentally friendly and chemically safe solutions. Consequently, impregnants with increased durability and multifunctional systems are being developed; these, in addition to fire resistance, also provide other functional properties, such as water repellency, antistatic properties, and stain resistance.

Another important goal is to replace traditional flame retardants with more environmentally friendly alternatives that are free of halogens and toxic phosphorus compounds, and yet retain their properties even after repeated washing. Among other solutions, biopolymers, nano-ceramic protective coatings and enzymatic modifications are used. The introduction of nanoparticles (e.g., graphene, carbon nanotubes) increases fire resistance without negatively affecting the comfort of use, flexibility, or aesthetics of the fabric.

The research will focus on issues related to the durability of modifications, i.e., ensuring that the fire-resistant properties are durable and do not degrade under the influence of external factors such as washing, environmental factors, and chemical or biological factors. Integrated research methods combining microstructural, spectroscopic, and thermal analyses are also becoming increasingly important, as they allow for a better understanding of the mechanisms of impregnation degradation and the development of more effective methods for their preservation.

It should be emphasised that the modification of materials must also take into account elements that are important from the point of view of application. One of the areas where modern fire-resistant materials are an important element of safety is fire protection, for which, in addition to fire retardancy, increased comfort, minimisation of health risks (carcinogens), durability, and resistance to chemical and microbiological factors are also important. In case of fabrics used in households, ease of cleaning, colour scheme, and durability will be important factors. Specific requirements must be met by materials intended for use in healthcare, such as in hospitals and medical clinics, for which requirements include self-cleaning, resistance to disinfectants, and antibacterial properties.

In addition to non-flammability and performance parameters, it is imperative to take into account the life cycle of the material being developed. In the era of climate change and growing public awareness, researchers and manufacturers should consider aspects such as the following:

− The availability of substrates;
− The possibility of using waste to produce, for example, flame retardants, modifiers, and substances that reduce energy consumption;
− Reductions in energy and water consumption;
− Supporting a circular economy;
− Reduction in waste.

Sustainable development also includes methods that enable the recycling of developed materials. Defective products and unused substrates generated during production should be subjected to neutralisation or recycling processes in order to minimise environmental and production costs.

The development of durable and multifunctional coatings supports a circular economy by reducing the need for frequent material replacement. Promoting sustainable sourcing and responsible practices enhances the long-term durability of products, and relying on green chemistry principles and safer chemical processes can contribute to reductions in environmental and health risks. Cost-effectiveness and integration with intelligent coating, joining, and quality monitoring technologies are also important development directions in the area of materials.

## 9. Conclusions

The durability of fire-resistant fabric impregnation is a key factor determining the effectiveness of protection in real-world conditions. Despite significant technological advances, modern flame-retardant methods still require improvement in terms of resistance to washing, dry cleaning, high temperatures, and UV radiation.

A review of the literature indicates that combining modern chemical methods (e.g., sol–gel, nanocomposites) with appropriate fibre surface modification may be a promising direction for further research into improving the durability and stability of fire-resistant fabrics. Substances derived from waste/biowaste or nanosubstances are used increasingly often in the production of modern fire-resistant materials. Due to their specific properties, such substances allow better fire resistance parameters to be achieved.

The development of fire-resistant fabrics is moving towards materials that are not only safe and effective, but also durable and environmentally sustainable, meeting other requirements of the areas in which they are used. Due to the specific nature of the area in question, the fire-resistant materials must also have other dedicated properties; this requires modifications considering not only fire resistance, but also, for example, self-cleaning, comfort of use, and chemical safety.

## Figures and Tables

**Table 1:** Number of publications, with associated citations, discussing the durability of fire-resistant fabrics, and the resistance of fire-retardant fabrics used by firefighters, published from 2016 to 2024 (based on Web of Science data).

Year	Durability of Fire-Resistant Fabrics	Resistance of Fire Retardant Fabrics Used by Firefighters	Citations
2025	1	1	27
2024	2	2	56
2023	0	1	53
2022	1	2	60
2021	0	1	87
2022	0	0	51
2019	3	0	38
2018	1	0	10
2017	2	0	2
2016	3	0	0

**Table 2:** Examples of fabric flame retardant methods.

Modification Technique	Characteristics	Ref.
Fiber modification	The flame retardant is added during the mixing and/or spinning processes; e.g., melamine polyphosphate (MPP) and modified melamine cyanurate (MCA) were added to the cellulose solution to produce flame-retardant Lyocell fibres, Lyocell-MPP and Lyocell-MCA, respectively, by dry and wet spinning methods.	[29]
Yarn modification	Modification takes place during yarn formation, e.g., by mixing flame-retardant fibre with ordinary fibre or by applying a flame-retardant agent to the surface of the yarn; e.g., between 100% cotton fabrics with a warp and weft yarn thickness of 28.4 Ne and flame retardants with a density of 600 g/L; during testing the absorption coefficient increased by 90%.	[30]
Dip baking	A flame retardant is dissolved or dispersed in a solvent—the fabric is passed through the solvent, and the flame retardant is rapidly absorbed into the fabric via rollers, and is then fixed on the fibre surface by high-temperature calcination; e.g., ethanol (C_2_H_5_OH) as solvent and 9,10-dihydro-9-oxa-10-phosphaphenanthrene-10-oxide (DOPO, flame retardant) resulted in a cotton fabric with a SiO_2_-KH570-DOPO hybrid solution.	[31]
Coating method/Spray method	The flame retardant is applied to one side of the fabric and then fixed by drying; e.g., modified keratin was applied to cotton fabric and then dried at 40 °C for 30 min, 80 °C for 1 h, and 110 °C for 3 min.	[32]
Chemical grafting	This involves bonding the flame retardant to the molecular chain of the fabric via a covalent bond; e.g., biomass-derived taurine was incorporated into Lyocell fabrics.	[33]
Layer-by-layer self-assembly	Flame retardants are deposited layer-by-layer on the substrate surface after alternating impregnation or spraying of the textile; e.g., cotton fabrics were treated successively with protein and phytic acid (PA).	[34]
Plasma method	The fabric surface is activated for more effective bonding with the flame retardant; e.g., cotton fabrics were exposed to atmospheric pressure dielectric discharge (APDBD) plasma and then treated with flame retardants (FR) using the pad–dry–cure method.	[35]

**Table 3:** Examples of methods used for verifying the quality of materials used in special firefighting clothing.

Parameter	Verification Method	Standards/Tests
Mechanical resistance of fabrics and seams	Abrasion resistance: tests to assess how well the material withstands prolonged contact with rough surfaces	[57,58,59,60,61]
	Tensile strength: determines the maximum force a fabric can withstand before breaking	[56,62]
	Tear strength: tear tests assess how easily an existing cut will enlarge under a load	[55,63]
	Seam strength: seams must hold the layers together under a load and not come apart	[8,64]
	Resistance to pilling: assesses the tendency of the fabric surface to form balls (pills) when rubbed	[65,66,67,68]
	Resistance to puncture: although not always required by fire safety standards, it is sometimes tested as an additional factor—e.g., resistance to puncture by a sharp object (test using a spearhead and force measurement)	[69]
Thermal resistance and heat protection	Resistance to flame (flammability test): the most basic test is a ‘small flame test’, which considers burning time after removal of the flame (afterflame time), glowing time (afterglow), length of charring/hole, occurrence of melting and dripping	[6,51]
	Resistance to thermal radiation (method B, for fabrics)	[53]
	Resistance to convective heat	[52]
	Resistance to contact heat	[70]
	TPP (Thermal Protective Performance) RPP (Radiant Protective Performance) HTI, RHTI, time to second-degree burns Thermal resistance of materials (stability)	[71]
Resistance to environmental and chemical factors	Resistance to UV rays and sunlight Resistance to rain, frost, and weather conditions	[71,72,73,74]
	Resistance to chemicals typically found in firefighting operations Accelerated climatic ageing	[71]
	Impact of fire pollutants Resistance to washing and soaking	[8,72,73,74,75,76,77]
Long-lasting comfort of use	Vapour permeability/water vapour resistance: measured, for example, as Ret (Resistance evaporative thermal) Thermal insulation (thermal resistance)	[78]
	Stiffness, elasticity Weight and water absorption Ergonomics versus wear	[79]

**Table 4:** Comparison of cleaning methods used for flame-resistant fabrics.

Method	Advantages	Disadvantages	Impact on the Durability of Flame Resistance
Water cleaning	Widespread availability, low costs	Fiber degradation, leaching of flame retardants	Moderate/highly negative
Dry cleaning	Gentle on fibres, absence of swelling	Risk of damage with improper solvents	Low
Low-water technologies	Controlled pH, gentle process	Higher equipment costs	Low/moderate
Layer-by-layer (LbL) coatings	Very high wash durability	High cost	Very low
Nanotechnology	High stability of flame retardancy	Research phase	Very low

## Data Availability

No new data were created or analyzed in this study. Data sharing is not applicable to this article.
